# Hepatitis B Virus (HBV) Subviral Particles as Protective Vaccines and Vaccine Platforms

**DOI:** 10.3390/v12020126

**Published:** 2020-01-21

**Authors:** Joan Kha-Tu Ho, Beena Jeevan-Raj, Hans-Jürgen Netter

**Affiliations:** 1 Victorian Infectious Diseases Reference Laboratory (VIDRL), Melbourne Health, The Peter Doherty Institute, Melbourne, Victoria 3000, Australia; Joan.Ho@mh.org.au (J.K.-T.H.); Beena.JeevanRaj@mh.org.au (B.J.-R.); 2 Royal Melbourne Institute of Technology (RMIT) University, School of Science, Melbourne, Victoria 3001, Australia

**Keywords:** hepatitis B virus, surface (envelope) antigen, sub-viral particle, virus-like particle

## Abstract

Hepatitis B remains one of the major global health problems more than 40 years after the identification of human hepatitis B virus (HBV) as the causative agent. A critical turning point in combating this virus was the development of a preventative vaccine composed of the HBV surface (envelope) protein (HBsAg) to reduce the risk of new infections. The isolation of HBsAg sub-viral particles (SVPs) from the blood of asymptomatic HBV carriers as antigens for the first-generation vaccines, followed by the development of recombinant HBsAg SVPs produced in yeast as the antigenic components of the second-generation vaccines, represent landmark advancements in biotechnology and medicine. The ability of the HBsAg SVPs to accept and present foreign antigenic sequences provides the basis of a chimeric particulate delivery platform, and resulted in the development of a vaccine against malaria (RTS,S/AS01, Mosquirix^TM^), and various preclinical vaccine candidates to overcome infectious diseases for which there are no effective vaccines. Biomedical modifications of the HBsAg subunits allowed the identification of strategies to enhance the HBsAg SVP immunogenicity to build potent vaccines for preventative and possibly therapeutic applications. The review provides an overview of the formation and assembly of the HBsAg SVPs and highlights the utilization of the particles in key effective vaccines.

## 1. Introduction

Hepatitis B is globally one of the most common infectious diseases in humans, which is associated with significant morbidity and mortality. Approximately 2 billion people worldwide have been infected with hepatitis B virus (HBV) and approximately 257 million people live with chronic HBV infections. An estimated 887,000 persons died in 2015 from acute or chronic consequences of hepatitis B [1,2,3,4]. The ability of the HBV structural proteins, including the hepatitis B surface (envelope) proteins (HBsAg) to assemble into non-infectious sub-viral particles (SVPs), allows the generation of highly organized particles displaying neutralizing epitopes that promote protective immune responses against the parent virus. The approval of the recombinant hepatitis B vaccine Recombivax HB (Merck Sharp and Dohme) in 1986, based on HBsAg SVPs and produced in the yeast *Saccharomyces cerevisiae*, was the first developed vaccine using recombinant DNA technology. The recombinant vaccine, together with the recombinant products, human insulin (licensed 1982), human growth hormone (licensed 1985), and alpha interferon (licensed 1986), demonstrated the capability of biotechnological approaches to generate innovative medicines [1]. The ability to accept foreign antigenic sequences into the SVP structure can provide the basis for the development of delivery platforms for targeted medically relevant sequences, as in the case of the RTS,S/AS01 (Mosquirix™) vaccine against malaria. The antigenic components of Mosquirix™ are chimeric SVPs containing HBsAg proteins fused to a *Plasmodium falciparum*-specific circumsporozoite (CS) polypeptide [5,6,7]. The design and generation of chimeric SVPs holds enormous potential in the treatment of infectious diseases, for which there are no effective vaccines [8,9,10].

## 2. Hepatitis B Virus, Classification, and Gene Products

**Figure 1:**
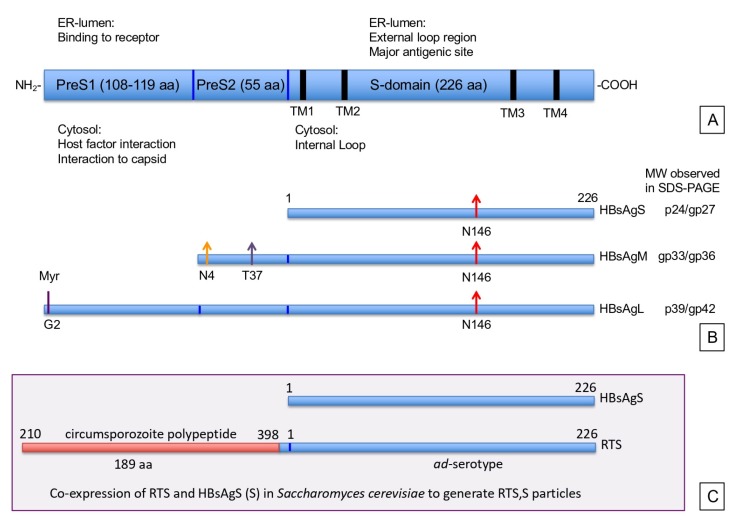
The surface (envelope) proteins (HBsAg) of hepatitis B virus (HBV): (**A**) The open reading frame encoding the complete hepatitis B surface antigen is depicted. The domain organization of preS1, preS2, and S, with the number of amino acids (aa) of the individual domains are specified. The four transmembrane regions (TM1–4) are indicated by the thick black lines. The function of the different domains in relation to their orientation towards the lumen of the endoplasmic reticulum (ER) or cytosol is indicated. (**B**) The individual HBsAg open reading frames for the small (HBsAgS), middle (HBsAgM), and large (HBsAgL) proteins, and their post-translational modifications are shown. The size of the HBsAgS protein is indicated by the number of amino acids. Arrows represent the utilized glycosylation sites. Red arrows mark asparagine 146 (N146) in the S-domain. Orange and purple arrows represent the N-4 and threonine 37 (T37) respectively, in the preS2 domain. The glycine residue at position 2 (G2) of the preS1 domain, indicated by a purple line, is myristolated. The observed molecular weights (MW) of the glycosylated (gp) and non-glycosylated proteins (p) separated under reducing conditions on a SDS-PAGE are indicated on the right. (**C**) Design of the RTS,S vaccine that is produced by co-expressing HBsAgS (aa 1–226) and the chimeric protein that is a fusion of the circumsporozoite polypeptide (210–398 aa) to the N-terminus of HBsAgS, including 4 aa from the preS2 domain.

HBV is a hepatocyte-tropic virus and is assigned to the family of hepatitis DNA viruses, *Hepadnaviridae* [11,12,13]. HBV is divided into 10 main genotypes, A–J, which differ by more than 8% at the nucleotide level [14,15]. The HBV genome has a size of approximately 3.2 kilobases (kb), and is represented by a relaxed circular, partially double-stranded DNA (rcDNA), which is delivered to the nucleus of the host cell and converted into a covalently closed circular DNA (cccDNA) molecule [12]. The cccDNA represents a non-integrated stable episome and forms the template for all viral RNA transcripts. In the absence of an origin of replication site required for DNA-dependent DNA amplification, one of the viral transcripts, the pre-genomic RNA (pgRNA), serves as the template for replication to generate rcDNA via reverse transcription [12]. HBV contains four open reading frames (C, P, S, and X) and encodes seven proteins (polymerase, X protein, HBcAg, HBeAg, HBsAgL, HBsAgM, and HBsAgS). The polymerase is essential for several steps in the replication pathway through its reverse transcriptase, RNaseH, and priming activities. The X protein supports efficient infection and replication in vivo [11,12,16]. The core protein (HBcAg) constitutes the subunit of the viral capsid and is essential for the formation of virions. The e-antigen (HBeAg) is derived from the pre-core protein by proteolytic processing and is not part of the viral capsid. It is involved in modulating the host immune response against HBV and represents an important serological marker [11,12,13]. The virus encodes for three related surface (envelope) proteins (HBsAg) that share a common S-domain. They are translated from different in-frame start codons and hence are distinguished by their N-terminal extensions. The small HBsAg (HBsAgS) comprises only the S-domain with a size of 226 amino acids (aa), the middle HBsAg protein (HBsAgM) has an N-terminal extension of 55 aa (pre-S2 domain), and the large HBsAg (HBsAgL) has an additional extension of 108 or 119 aa (preS1-domain) depending on the genotype [17] (Figure 1A,B). In addition to the classification by genotypes, HBV is distinguished by four main serotypes based on the reactivity against HBsAg. All genotypes have a common serotypic reactivity against a major antigenic site called the “a”-determinant, but further express two mutually exclusive allelic antigenic determinants “d” or “y” and “w” or “r” [18,19,20]. The antigenic determinants of HBsAg are located in an exposed loop region of the S-domain. HBsAg and antibodies against HBsAg (anti-HBs) are important serological markers. The loss of HBsAg and seroconversion to anti-HBs antibodies are a sign of immunity and recovery from acute or chronic hepatitis B [13].

Characteristic of a HBV infection is the generation of a large quantity of HBsAg SVPs and filaments devoid of capsid and of the viral genome. SVPs exceed the presence of infectious virions in host sera by a factor between 10^2^ and 10^5^ [17,21,22,23,24]. SVPs are predominately composed of HBsAgS, and their presence in the sera does not seem to interfere with HBV particle entry into hepatocytes, suggesting that SVPs represent decoys by binding to virus-neutralizing antibodies [25]. HBsAgS SVPs share important immunological determinants with the mature virus, and therefore, SVPs derived from patient serum or recombinant SVPs represent effective immunogens for the induction of a protective immune response [26,27,28]. Vaccinated individuals develop antibodies targeting the “a”-determinant region, which provides protection against the infection of all HBV serotypes [20,29]. The discovery and characterization of “empty” genome-free virions containing HBsAg and capsid is reviewed by Hu and Liu, 2017 [30].

## 3. Role of HBsAg in HBV, Filament, and SVP Formation

An essential step in the formation of virions, filaments, or SVPs is the cotranslational insertion of the HBsAgS protein into the membrane of the endoplasmic reticulum (ER) with a short luminal exposed N-terminal sequence, two transmembrane regions separated by a cytosolic loop, and a luminal domain, followed by a hydrophobic C-terminal region. The luminal domain corresponds to the external loop region of the S-domain, which contains multiple epitopes, including the immunodominant “a”-determinant region that is common to all HBV genotypes, and the allelic antigenic determinants “d/y” and “w/r” [17,31]. A conformational heparan sulfate binding site also overlaps with the “a”-determinant region and is essential to infectivity [32].

**Figure 2:**
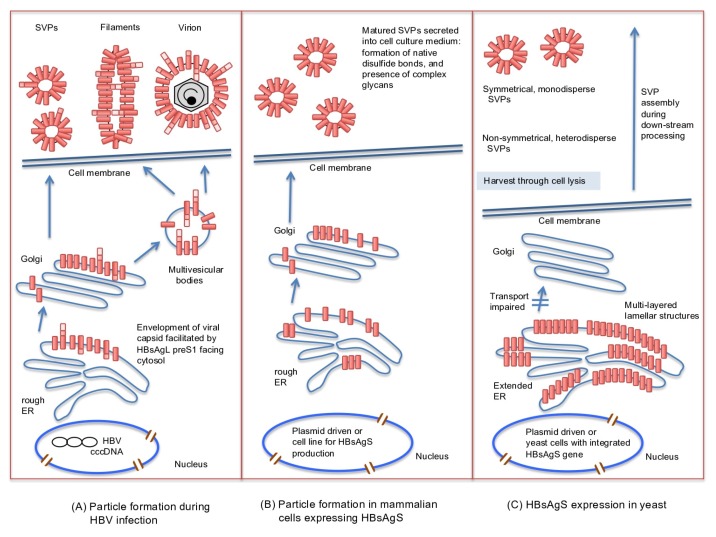
Illustration of HBsAg protein synthesis and assembly pathways during a natural infection (**A**), HBsAgS expression and assembly in mammalian cells in the absence of other viral gene products (**B**), or expressed in yeast (**C**). During a natural infection, virions and filaments are formed by budding from multivesicular bodies. The spherical subviral particles (SVPs) are produced and secreted through the endoplasmic reticulum (ER)-Golgi complex. The HBsAg subunits of the virions, filaments, and SVPs form intra- and intermolecular disulfide bonds, and are partially glycosylated (not shown) (**A**). Expression of the HBsAgS gene in mammalian cell lines leads to the formation of SVPs exclusively composed of HBsAgS permitting the formation of disulfide bonds and partial glycosylation (not shown) (**B**). HBsAgS protein expressed in yeast accumulates in the ER, causes an extended ER and forms multilayered lamellar structures. HBsAgS protein complexes are harvested from the cell lysate, and SVPs are assembled during down-stream processing (**C**). cccDNA: covalently closed circular DNA.

HBV virion formation depends on the presence of the viral capsid containing rcDNA and the HBsAg proteins for envelopment. The preS1 region of HBsAgL is essential for the assembly of infectious HBV particles by interacting with the capsid [17,33], possibly contributes to the binding to a proteoglycan attachment site [34,35], and is required for binding to the hepatocyte entry receptor, sodium taurochlorate cotransporting polypeptide (NTCP) [36,37]. Chaperones of the heat shock protein (hsp) 70 family facilitate the interaction of the HBsAgL preS1 domain with the capsid by retaining the preS1/preS2 sequence in the cytosolic (internal) orientation at the ER [38,39,40]. Virion secretion depends on host factors of the endosomal sorting complex required for transport (ESCRT) and sorting into late endosomal multivesicular bodies (MVBs), and finally, release of its intraluminal content at the hepatocyte surface [41,42,43]. The mature HBV virions are spherical, enveloped particles with a diameter of 42 nm with an inner capsid of 22 nm in diameter [11,12,13]. Due to the elevated presence of HBsAgL in virions and filaments compared to the SVPs, filaments seem to follow the secretion pathway taken by the virions (Figure 2) [44]. The filaments have a width of approximately 20 nm and are variable in length [12,23,45].

In contrast to the formation of infectious particles, the formation of SVP does not depend on the preS1 domain. HBsAgS proteins have the ability to assemble into secretion-competent SVPs and are composed solely of envelope proteins, lipids, and glycans. Contrary to virion secretion, SVP assembly follows the constitutive secretory pathway of the host cell for release [43,44]. The non-infectious SVPs are 17 to 25 nm in diameter. SVPs isolated from HBV carriers show spike-like features protruding from the surface similar to the surface projections observed on filaments and infectious virions (Dane particles) [24,45,46,47,48]. An alternative structure for recombinant SVPs expressed in transgenic mice has been proposed to possess an octahedral symmetry [49]. Mammalian cell lines expressing HBsAgS in the absence of any other viral component secrete SVPs, which are morphologically indistinguishable from serum-derived SVPs (Figure 2) [50,51,52,53,54]. Thus, particle morphogenesis substantially differs between SVPs and virions, but they have identical antigenic structures due to the S-domain, which is encoded by all HBV envelope proteins (Figure 1A,B).

### 3.1. Topology of HBsAgS

**Figure 3:**
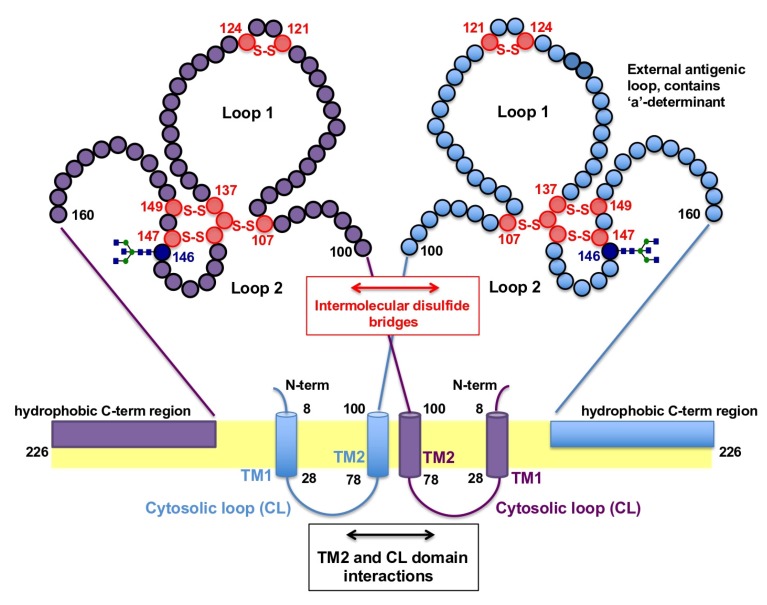
Proposed model for the formation of the HBsAgS homodimer based on Suffner et al. [57]. Dimerization of two HBsAgS monomers represented in purple and blue is facilitated by interactions of the transmembrane domains (TM2), cytosolic loops (CL) and intermolecular disulfide bridges (S-S) between cysteine residues (red circles) in the external loop region. The facultative *N*-glycosylation site at position N146 is indicated (dark blue). The orientation of the hydrophobic C-terminal region is illustrated in a simplified form.

The correct folding of HBsAgS depends on two topogenic signal sequences, which determine the orientation of the S-domains in relation to the lipid layer. The insertion of the HBsAgS N-terminus into the ER membrane requires the presence of the topogenic N-terminal signal sequence 1 (transmembrane region 1, TM1, aa 8–22), which is not proteolytically cleaved by the host’s peptidases, and allows the translocation of the N-terminus across the ER membrane. The second internal topogenic transmembrane sequence (TM2), which is located between aa 80 and 98, supports the translocation of flanking C-terminal sequences and serves as an anchor to hold the sequence in the membrane [17,55,56]. Both topogenic signal sequences are required for the correct folding of HBsAgS, resulting in the formation of a cytosolic loop (aa 23 to 79), and a loop reaching into the ER lumen (aa 99 to approximately 155), followed by a proposed amphipathic helix (aa 156 to 169) and the hydrophobic C-terminal region (aa 170 to 226) embedded in the ER membrane (Figure 1A and Figure 3) [57]. The luminal loop region of the S-domain is located at the external surface of the mature SVPs and also infectious virions, and harbors the major HBsAg protein epitopes (“a”-determinant, and “d/y”, “w/r” determinants) [13]. The hydrophobic C-terminal region (aa 170 to 226) possibly contains two additional transmembrane regions, as indicated by topological models [58,59]. The presence of transmembrane passages is supported by experimental data suggesting that the C-terminal sequences are exposed at internal and external surfaces. The sequence between residues 196 and 201 is important for packaging of hepatitis delta virus (HDV), a satellite of HBV, and therefore expected to be accessible to facilitate the interaction with the HDV ribonucleoprotein complex [60]. A second site between residue 178 and 186 is targeted by an anti-HBs monoclonal antibody, indicating surface exposure [61].

HBsAg proteins contain 14 cysteine (cys) residues located in the S-domain, which are highly conserved among different HBV genotypes; the preS1 and preS2 domains do not contain additional cysteine residues [12,17]. Eight cysteine residues are located in the external loop region, forming disulfide bonds which are important for the integrity of the major antigenic determinants [62,63], such as cys-107 in the external loop for retaining the “a”-determinant specific antigenicity [64]. The identification of HBsAgS oligomers and polymers by electrophoresis under non-reducing conditions suggests that the disulfide bonding is heterogeneous, consistent with the finding that only a fraction of HBsAgS subunits are exclusively linked by disulfide bonds formed between cys-121 and cys-147 (Figure 3) [62,63,65]. Reduction or absence of intermolecular disulfide bridges interferes with the native HBsAgS antigenicity but allows SVP formation [62,65,66].

The S-domain shared by the HBsAg proteins harbors an *N*-glycosylation site at position asparagine-146 (N146), which is partially utilized by the oligosaccharyltransferase [17,67], and hence, HBsAgS proteins are synthesized as unglycosylated p24 and *N*-glycosylated gp27 versions (Figure 1B). HBsAgS p24 and gp27 have identical transmembrane topologies and dimerize without preference for a specific pairing and form heterologous dimers with HBsAgM and HBsAgL [66]. Synthesis and secretion of HBsAgS SVPs do not depend on *N*-glycosylation at position N146 in the external loop region, in contrast to the formation and release of infectious HBV particles [67,68,69,70].

### 3.2. Topology of HBsAgM

The middle HBsAg protein has the same transmembrane topology as HBsAgS, and hence is partly glycosylated at N146 in the S-domain. Translocation of the preS2 domain of HBsAgM is mediated by the topologic signal TM1 located in the S-domain [71]. The translocation event across the ER membrane into the lumen allows the *N*-glycosylation of the asparagine-4 (N4) residue in the preS2 domain, which is always glycosylated [72], resulting in glycoproteins with a molecular weight of 33 kDa and 36 kDa (Figure 1B). In addition to the *N*-glycosylation site in the preS2 domain, the preS2 domain can be partially *O*-glycosylated, depending on the genotype and the presence of the threonine (T37) (Figure 1B) [73]. Similar to HBsAgS, HBsAgM proteins assemble into SVPs and can be secreted independently from other viral proteins [74,75,76,77]. HBsAgM, however, is not essential for virion morphogenesis and infectivity [78].

### 3.3. Topology of HBsAgL

The large HBsAg protein adopts two distinguished transmembrane topologies facilitating an internal or external location of the preS1/S2 domain (Figure 1A) [17,79,80,81]. The internal orientation allows preS1/S2 to interact with the viral capsid, a critical step in viral morphogenesis. The preS2 region of the HBsAgL protein serves as a possible spacer to facilitate conformational changes of the preS1/S2 domain [17,81,82]. During the maturation process, the preS1/S2 domain is translocated to adopt an external orientation, which is essential for virus attachment to the host cell through a specific interaction with heparan sulfate proteoglycan [34,35] and binding to the entry receptor [36,37]. In addition, the preS1 domain is myristoylated at the N-terminus, which is required for efficient HBV entry into hepatocytes (Figure 1B) [83,84]. The potential *N*- and *O*-glycosylation sites in the HBsAgL preS1/S2 domain are not utilized due to the cytosolic orientation of the preS1/preS2 domain after translation. HBsAgL molecules have molecular weights of 39 kD (p39) or 42kD (gp42) depending on the glycosylation status at position N146 in the S-domain (Figure 1B). Expression of HBsAgL in mammalian cells in the absence of HBsAgS and HBsAgM does lead to particle formation, but not secretion, and it is retained in post-ER and pre-Golgi compartments [85]. HBsAgL causes a dose-dependent inhibition of particle release if co-expressed with HBsAgS [86,87].

Taken together, the S-domain is shared by the HBV envelope proteins and defines the backbone of the particle due to the presence of the topogenic transmembrane regions, glycans at position N146, cysteine residues to form inter- and intra-molecular disulfide bonds, and the external loop region. The correct folding of the external loop defines the HBsAg-specific antigenic determinants.

## 4. Biochemical Properties of SVPs

The proportion of the HBsAgL, -M, and -S proteins differ between Dane particles, filaments, and SVPs generated during a natural infection. It is estimated that the envelope of Dane particles contains HBsAgL, HBsAgM, and HBsAgS at a ratio of approximately 3:2:5, and filaments at a ratio of 1:1:4. SVPs contain less HBsAgM compared to filaments with trace amounts of HBsAgL [46,83,88]. HBsAgS SVPs have a molecular weight of 2–4 × 10^6^, and are composed of protein (75% by weight), carbohydrates (in form of glycoproteins) and lipids (25% by weight) [89]. Approximately 100 HBsAgS proteins assemble with lipids into lipoprotein particles, fifty HBsAgS dimers were identified in SVPs purified from sera of transgenic mice [49,89,90]. Three different regions of the S-domain contribute to the oligomerization of the HBsAg proteins, the cytosolic loop, TM2, and the luminal loop (Figure 3) [57]. The SVPs are compact particles with a reported density of 1.21 g/mL in caesium chloride (CsCl) compared to a density of infectious virions between 1.24 and 1.26 g/mL [23,91,92]. The compact structure of the SVPs is due to the large number of intra- and inter-molecular disulfide bonds within and between the S-domains of the individual HBsAg subunits [62,65,90,93,94,95]. Kinetic studies demonstrated that disulfide-linked HBsAgS dimers are formed in the ER, then the immature particle precursors are transported to a post-ER, pre-Golgi compartment, which excludes the enzyme “protein disulfide isomerase” and allows the formation of HBsAgS oligomers [96]. Intracellular HBsAgS particles contain high-mannose oligosaccharide chains, and after secretion, SVPs contain complex oligosaccharide chains with terminal sialic acid *N*-acetylglucosamine residues representing glycosylation patterns conforming with the HBsAg movement from the ER through the Golgi cisternae [12,67,97].

Cryo-EM studies and biophysical analyses of the SVPs produced in cell culture or purified from sera of transgenic mice demonstrates a tight HBsAgS protein–lipid interaction. The lipid composition of HBsAgS SVPs purified from the plasma of several HBV carriers showed that phospholipids, in particular, phosphatidylcholine is a major lipid class; with palmitic, stearic, oleic, and linoleic acids being the major fatty acid components [89]. Consistently, SVPs produced in human hepatoma cell lines predominantly contain phospholipids, with phosphatidylcholine being the dominant component [98]. The tight protein–lipid interaction restricts lipid movement suggesting that the lipids are not aligned in a typical bilayer structure. HBsAg particles seem to contain the lipids in an unusual arrangement, with the lipids being closely intercalated with the proteins, located on the particle surface, and are hence likely arranged in a lipid monolayer [99,100]. HBsAg proteins contain a high content of alpha helices (45%–52%), which are lipid-associated, and provide an arrangement which allows the disposal of the loop regions in the particle interior or on its surface [100,101]. Assessing yeast (*Hansenula polymorpha*)-derived SVPs, the particles have an ordered and rigid lipid interface, possibly organized as a phospholipid monolayer, with a hydrophobic and fluid inner core. HBsAgS proteins penetrate into the lipid core, with parts of the protein protruding from the particle surface [89,102,103]. The lipids contribute to the antigenic activity of HBsAg particles [100], likely by stabilizing the proper helical structure of the HBsAg proteins and the conformation of their hydrophilic region, which contains the antigenic site. Removal of lipids decreases the helical content and reduces the antigenic activity of the particles [100]. S-domain antigenic structures seem to be strongly impacted by the lipid–protein interface, which defines the formation of alpha-helical structures and accommodates the arrangement of proper disulfide bonding patterns and the correct folding of HBsAg.

## 5. The First Vaccine Generation Against HBV: Vaccine Derived from Patient Plasma

The first vaccine against HBV was based on the unique approach to purify the HBsAg immunogen directly from the blood of asymptomatic HBV carriers [104]. The identification of the HBsAg as an important immune target was based on observations that anti-HBs human immunoglobulins conferred passive protection against hepatitis B [105,106]. Consistently, active immunization studies with HBsAg protected chimpanzees from hepatitis B [29,107,108], and clinical studies with recipients of blood transfusion indicated that patients who developed anti-HBs were less likely to develop hepatitis [109]. Patients on a renal dialysis unit and staff were less likely to acquire hepatitis if they had anti-HBs antibodies [110]. The first reported vaccination against hepatitis B was performed with a diluted, heat-inactivated HBsAg-positive serum in children, then the children challenged with infectious HBV, resulting in an incomplete but considerable protection [111]. For the generation of a proper vaccine, methodologies were developed to purify HBsAg using isopycnic banding and rate-zonal separation [26,27,112,113], affinity columns [114], adsorption onto colloidal silicate and desorption, differential polyethylene glycol (PEG) precipitations and gel filtration [27]. To minimize the risk of infections due to the presence of hepatitis B virions, the vaccine preparations were inactivated with formalin [112,114]. Plasma was from “ad” and “ay” donors, the SVPs mixed, then adsorbed onto aluminium hydroxide, or used in the absence of an adjuvant [26,27]. The pilot vaccines demonstrated that the plasma-derived SVPs induced anti-HBs antibody responses in different animal models [26]. Safety testing in chimpanzees, which are susceptible to HBV infection, did not provide any evidence of hepatitis in chimpanzees, which had received the inactivated plasma-derived SVP vaccine. Vaccinated chimpanzees were protected from a challenge with infectious HBV and did not show any indication of a hepatitis B infection [26,108].

The use of the first plasma-derived vaccines, Heptavax-B (Merck Sharp and Dohme, MSD) and Hevac-B (Institute Pasteur) provided good protection rates, and they were safe [114,115,116,117]. Depending on the purification and inactivation procedure, the composition of the plasma-derived HBsAg SVPs can vary, and they may or may not contain small quantities of HBsAgM, providing the preS2 domain in addition to HBsAgS. The Hevac-B vaccine contained 1%–2% HBsAgM. In contrast, the Heptavax-B vaccine did not contain preS2 proteins due to a treatment step with proteases, which however did not interfere with HBsAg-specific antigenicity [118,119,120]. Similar serum-derived vaccines were then produced from various manufacturers, such as Hepavax-B (Green Cross, Korea), Hepaccine-B (Cheil, Korea), and GCC VAC (Green Cross Corporation, Osaka) (Table 1). Limitations given by the supply of human plasma from chronically infected patients, and the risk associated with human-derived products due to contaminating proteins and the potential presence of other pathogens transmitted by blood, in particular a non-A non-B hepatitis virus, confronted the use of human plasma-derived HBsAg SVP vaccines [121,122,123]. Safety concerns about products from human sources, together with the advances in recombinant DNA technology and biotechnology, led to the development of recombinant hepatitis B vaccines.

## 6. The Second Vaccine Generation Against HBV: Yeast-Derived Recombinant HBsAgS SVPs

The identification of HBsAgS as the major HBV envelope protein of plasma-derived HBsAg SVPs and encoding the major antigenic determinants prompted the expression of HBsAgS in mammalian cell lines and yeast [13]. Yeast cell strains (*Saccharomyces cerevisiae, Pichia pastoris, Hansenula polymorpha*) were developed which express HBsAgS in high quantities. HBsAgS SVPs are isolated from yeast cell extracts with a sedimentation rate and buoyant density similar to particles from human samples or expressed in cell culture [138,139,140]. Yeast-derived SVPs have reduced antigenic reactivity compared to SVPs derived from human plasma but immunization studies established that yeast-derived SVPs induce anti-HBs antibody responses, which provide protection of immunized chimpanzees following a challenge with HBV. Importantly, the yeast-derived HBsAgS SVP vaccine of the “adw” subtype conferred protection against HBV subtypes “adr” and “ayw” [28]. Clinical trials using different age groups, healthy individuals, and special target populations confirmed that the yeast-derived vaccine is highly immunogenic, and generated qualitative and quantitative anti-HBs antibody responses with a protective efficacy similar to the plasma-derived vaccines [141]. The vaccines achieved 99% seroprotection rates in healthy children and adolescents but approximately 5%–7% of the adult population are non-responders and the rate can increase to 70% in elderly persons and in special risk groups [13,129,136,142]. Also, genetically determined resistance may contribute to non-responsiveness to HBsAgS SVP vaccines [143,144,145]. At the molecular level, yeast-derived HBsAg SVPs are not *N*-glycosylated at the N146 position within the S-domain, in contrast to SVPs produced in mammalian cell lines and harvested from the cell culture medium, or isolated from the blood of chronic hepatitis B patients. HBsAgS expressed in yeast generate SVPs [146], but experimental evidence indicates that the SVP are not formed within the yeast cell and generated during the down-stream purification procedures. HBsAgS expression in *Pichia pastoris* showed that HBsAgS assembles at the ER into multi-layered lamellar structures [147]. Monitoring SVP assembly during the purification procedure demonstrated that particulate structures are formed after eluting HBsAg bound to colloidal silica. Irregular SVP-like structures were visualized, and morphological changes observed after pH adjustment (colloidal silica eluate pH 10.8 to 8.0), ion-exchange, and size-exclusion chromatography. The monodispersity improved after potassium thiocyanate (KSCN) treatment, also the SVPs have a more-fine structured surface [148,149,150]. The correct disulfide bonding is the molecular basis for the formation of native epitopes as probed with an anti-HBs antibody [151]. Lipid-containing SVPs undergo KSCN-induced maturation by the formation of intra- and inter-molecular disulfide bonds to generate fully disulfide-bonded SVPs, resulting in a decreased conformational flexibility of HBsAgS in the matured particles [149,152]. Restricted conformational flexibility is possibly required for the formation of native HBsAg-specific antigenic structures, which allows epitope recognition by anti-HBs antibodies, and importantly in eliciting neutralizing antibodies [149]. The particle size (diameter) of yeast-derived SVPs is consistent with the data obtained from mammalian-cell-derived SVPs, but the size distribution can vary depending on the SVP maturation level [148,153]. Yeast-derived SVPs have a high content of alpha-helical structures, and the movement of the HBsAgS proteins is restricted due to the tight association with the lipid membrane [102,154]. Similarly, the lipid composition is characterized by high levels of phospholipids, in particular, phosphatidylcholine consistent with mammalian cell-derived SVPs [154,155,156]. No significant differences in the anti-HBs response induced by the plasma-derived or yeast-derived hepatitis B vaccines was observed [123,157].

The widely distributed, yeast cell-derived hepatitis B vaccines, Engerix^®^-B (GlaxoSmithKline) and Recombivax HB™ (Merck Sharp and Dohme) use aluminium hydroxide or aluminium hydroxyphosphate sulfate as adjuvanting substances, respectively. Aluminium-based adjuvants are widely used and activate the inflammasome pathway [158,159,160]. The adsorption of HBsAgS SVPs on aluminium hydroxide is mediated by binding of the phosphate groups of the HBsAgS SVPs phospholipids with hydroxyl groups of aluminium hydroxide through a ligand-exchange mechanism [161,162]. After vaccine administration, the SVPs are eluted from the aluminium adjuvant upon contact with the interstitial fluid [163,164]. Adsorption of HBsAgS SVPs derived from *Hansenula polymorpha* on an aluminium gel followed by a mild desorption step using competing phosphate anions demonstrated that the conformation of the HBsAgS protein is retained, and consistently, no significant changes of the lipid core and lipid membrane surface of the SVPs were identified [165]. To improve immunization outcomes in adults at risk of a hepatitis B infection, HBsAgS SVPs synthesized in *Hansenula polymorpha* are formulated with a Toll-like receptor 9 (TLR 9) agonist, cytidine-phosphate-guanosine oligodeoxynucleotide (CpG-ODN) 1018 as an adjuvant (Heplisav-B™, Dynavax Technologies). TLR9 is a pattern recognition receptor of the innate immune system, which induces the production of cytokines such as interleukin-12 and interferon-alpha to stimulate the adaptive immune response [134,166]. Heplisav-B™ induced earlier seroprotection rates allowing a two-dose regimen compared to three doses required for the Engerix-B vaccine, but it caused more injection-site reactions [134].

The ability of HBsAgS subunits expressed in yeast cells to form SVPs during down-stream procedures, and to reproduce native antigenic structures, allowed the development of a highly successful preventative vaccine. The advancement of the vaccine adjuvant technology allowed new HBsAgS SVP formulations and demonstrated strategies to enhance the magnitude of the anti-HBs immune response with immediate practical applications

## 7. Third Generation Vaccine Concepts against HBV

The development of third-generation HBsAg vaccines providing the S-domain in combination with preS1 and/or preS2 sequences was directed by the objective to enhance the protective efficacy of the human plasma-derived vaccines (which consisted predominantly of HBsAgS subunits), and the second-generation recombinant yeast-derived vaccines (which consisted exclusively of HBsAgS subunits). The third-generation vaccines attracted interest to improve the immunization outcomes in persons who do not respond to the conventional HBsAgS vaccines [13]. The importance of the preS1 domain for viral entry and assembly makes it a potential target for vaccine development, anti-preS1 antibodies protected chimpanzees from HBV infection [167]. PreS1/S2 sequences provide additional B-cell epitopes to generate protective antibody responses [167,168,169] and may also serve as a T cell immunogen to overcome the non-responsiveness to the S-domain [170,171,172]. Small quantities of HBsAgL present in the HBsAgS vaccine induced significant T-cell activation measured as in vitro proliferation specific for the preS domain [172]. In addition, preS2 peptide vaccines protect chimpanzees against a challenge with HBV [169], and the use of SVPs with preS1 and/or preS2 sequences generated anti-HBs immune responses in mouse strains, which are non-responsive to the standard yeast-derived HBsAgS SVP vaccine [173].

For the generation of third-generation vaccines, different yeast expression systems and mammalian cell lines were utilized to synthesize SVPs composed of HBsAgM and HBsAgL proteins in the presence or absence of HBsAgS. Utilizing *Saccharomyces cerevisiae*, the expression of a modified HBsAgM in the absence of HBsAgS allowed the formation of SVPs. The modified HBsAgM protein (P31c) contains a deletion of six amino acids to make it resistant to trypsin-like proteases in *S. cerevisiae*. The HBsAgM-P31c proteins assembled into SVPs with a diameter of approximately 20 nm and retained HBsAg antigenicity [174,175]. Immunization studies in BALB/c mice and guinea pigs demonstrated that anti-HBs antibody titres can be induced comparable to the plasma-derived Heptavax-B vaccine (MSD). Also, anti-preS2 antibodies were detected in animals immunized with the HBsAgM-P31c SVP vaccine. In contrast to the yeast-derived HBsAgS vaccine, the HBsAgM-P31c SVPs are glycosylated with N- and O-linked glycans located in the preS2 domain [174,175], which may facilitate interactions with lectin receptors expressed by antigen-presenting cells. In an independent study, HBsAgM SVPs induced anti-S and anti-preS2 antibodies in healthy young adults but the anti-S response was lower than in the patient group who received HBsAgS SVPs, and hence, the HBsAgM vaccine failed to achieve the objective of inducing an early and strong anti-S and anti-preS2 immune response [176]. The HBsAgM-P31c SVPs, however, were used to formulate a new vaccine (TGP-943, Takeda) and demonstrated a protective effect in the chimpanzee model and also generated protective levels of anti-preS2 antibodies in humans [177,178]. Clinical studies demonstrated that the vaccine TGP-943 induced both anti-S and anti-preS2 antibodies, approximately 50% of non-responders became positive for either or both anti-S and anti-preS2 [131].

For the development of a HBsAgL-based vaccine, HBsAgL expression during the exponential *S. cerevisiae* growth phase generated high levels of HBsAgL but did not assemble into the typical 20–25 nm SVPs, but generated a polydisperse population of small (2–3 nm) and large aggregates (15–50 nm) [179]. HBsAgL was glycosylated by *N*- and *O*-linked glycans in the preS1/S2 domain indicating that HBsAgL accessed the lumen of the ER of the yeast cell and caused morphological changes in the ER compartment [179,180]. The presence of *N*-linked and *O*-linked glycans in the preS1/S2 domain of yeast-derived HBsAgL proteins is in contrast to preS1/preS2 of HBsAgL isolated from human plasma due to the cytoplasmic exposure of the preS1/S2 domain during the orderly and regulated process of virion morphogenesis [83,88,179,180]. Particle formation using *S. cerevisiae* could be rescued providing an N-terminal signal sequence, which possibly allows a correct entry into the secretory pathway, and after purification from the yeast lysate, spheres and filaments with a diameter of 23 nm were obtained, the length of the filaments was in the range of 40 to 120 nm, visualized by negative staining electron microscopy. HBsAgL proteins expressed in the absence of the signal sequence did not form such an ordered structure [181]. The visualization of the particles by atomic force microscopy (AFM) demonstrated a heterogeneous population of rugged spherical forms between 50 and 500 nm in diameter [182]. Immunization of mice with the yeast-derived HBsAgL SVPs elicited anti-S, anti-preS2, and anti-preS1 antibodies, and the effective dose (ED_50_) for anti-S and anti-preS2 antibodies were similar to those achieved with HBsAgM particles [182]. Using an alternative strategy, hybrid SVPs were generated in *S. cerevisiae* composed of HBsAgS and a modified HBsAgL (HBsAgL*). HBsAgL* contains a truncated preS1/S2 region with sequences relevant for the hepatocyte-binding site and immunologically important B- and T-helper epitopes but does not contain sites for proteolysis and the binding site for polymerized human serum albumin. The hybrid SVPs contained HBsAgS and HBsAgL* at a ratio of 75:25 [176]. The immunization of BALB/c mice with HBsAgL*/HBsAgS SVPs generated anti-S and anti-preS1 antibodies. The anti-S titers were similar to those found after immunization with Engerix B. Immunizations of African Green Monkeys (*Ceropithecus aethiops*) using HBsAgL*/HBsAgS SVPs induced anti-S, anti-preS2, and anti-preS1 antibodies [176]. Safety and immunogenicity studies in young, healthy adult persons, and in poor responders to hepatitis B vaccines demonstrated that the presence of the preS1/preS2 domain did not enhance the anti-S response compared to the control Engerix B vaccine (GSK) [183,184], in spite of the preS1 sequence present as a strong T-cell immunogen [170]. The HBsAgL*/HBsAgS vaccine induced anti-preS1 antibodies in a young, healthy adult person cohort, and possibly provides additional neutralizing activity [183].

With the availability of mammalian cell culture technologies, recombinant hepatitis B vaccines composed of SVPs have been developed containing the HBsAgS and HBsAgM (GenHevacB, Sanofi Pasteur Vaccins) and the additional HBsAgL subunits (Sci-B-Vac, VBI Vaccines; Hepacare, Medeva Pharma) (Table 1). Chinese hamster ovary (CHO) cells (GenHevacB, Sci-B-Vac) [120,136] or murine cells (C1271) (Hepacare, Medeva Pharma) [137] were used to generate the vaccines. The GenHevac B vaccine is composed of HBsAgS/HBsAgM SVPs at a ratio 80:20, and compared in a clinical setting to the human plasma-derived Hevac B vaccine, both vaccines induced antibodies to the HBsAg in >90% of the participants (497 persons in the age range of 18–40 years). Compared to the plasma-derived vaccine, the recombinant vaccine produced early and high levels of anti-preS2 antibodies, which may provide an additional advantage in prevention of a HBV infection [120,185].

For Sci-B-Vac, the complete HBsAg gene encoding HBsAgS, -M, and -L, including native promoter, enhancer, and poly(A) signal, were used to establish a producer CHO cell line, which contains more than 100 HBsAg coding copies/cell [173]. Protein analysis of the secreted SVPs revealed the presence of all three HBsAg proteins and its glycosylated isoforms (HBsAgS p24 and gp27; HBsAgM gp33 and gp36, HBsAgL p39 and gp42). Sci-B-Vac induced anti-S and anti-preS1 antibody responses in BALB/c mice, and also in mouse strains which are resistant to immunizations with HBsAgS SVPs and/or HBsAgM [136,173]. Sci-B-Vac demonstrated an excellent safety record in clinical studies, which included healthy individuals, children, and neonates. In comparison with yeast-derived hepatitis B vaccines, more than 50% of vaccinees receiving Sci-B-Vac developed earlier seroprotection against HBV [136]. Sci-B-Vac performed superior to yeast-derived HBsAgS vaccines in specific patient risk groups and provided vaccine boosts in persons with no or low response to preceding immunizations with the conventional yeast-derived HBV vaccine. Specific risk groups including patients with renal failure, with overweight and immune-suppressed patients responded with higher seroprotection rates compared to conventional yeast derived vaccines. The Sci-B-Vac vaccine is widely used in Israel and licensed in various countries [136].

## 8. HBsAgS SVPs as Platforms for Medically Relevant Antigenic Sequences

SVPs display an array of antigenic sequences to the innate immune system facilitating the subsequent activation of the adaptive system [8,10,186,187]. The ability to accept foreign inserts into the SVP structure provides the basis for advanced delivery platforms for medically relevant sequences, such as malaria antigens. Chimeric SVPs can be constructed from viral capsid proteins, such as capsids from HBV, human papilloma virus, and Qβ phage that have been re-engineered to express foreign antigenic sequences at a high antigenic density [8,187]. Similarly, SVPs derived from the HBV envelope assemble into highly compact lipid-containing particles, and have been exploited as carrier platforms for foreign antigenic sequences by introducing N- or C-terminal extensions [188,189,190,191], N-terminal extensions in addition to substitutions of the HBsAgS N-terminal sequence [192], by replacing the HBsAgM preS2-domain [193,194], by insertions into the external loop region including replacing antigenic determinants [195,196,197,198,199,200,201], or by replacing HBsAgS-specific cytotoxic T lymphocyte (CTL) epitopes [202] (Table 2). The insertion of a poliovirus-specific epitope with a length of 11 amino acids into the external loop region of HBsAgS allowed the expression of the chimeric, assembly and secretion competent HBsPolioAg proteins in a mouse cell line [201,203]. The chimeric SVPs contained glycosylated and non-glycosylated HBsPolioAg subunits and formed particles with 22 nm in diameter, similar to wild-type HBsAgS proteins. HBsPolioAg SVPs were used in mouse immunization studies and induced anti-poliovirus peptide-specific antibodies with neutralizing activity and a low level of anti-HBs antibodies, possibly due to a partial loss of HBsAg-specific antigenicity. The co-expression of both wild-type HBsAgS proteins and HBsPolioAg generated hybrid SVPs composed of both proteins, which facilitated the induction of anti-HBs and anti-poliovirus epitope antibodies [203]. Similar studies inserting heterologous B-cell epitopes in an exposed site in the external loop region reduced HBsAg-specific antigenicity depending on the length of the insert, but the recombinant proteins retained the ability to induce anti-HBs antibodies [195,196,198,204]. Chimeric SVPs composed of subunits distinguished by the number of inserted epitope repeats from the *Plasmodium falciparum* circumsporozoite (CS) protein demonstrated that the CS epitope number influenced the activity of the anti-CS epitope antibodies. The effect of the epitope-specific density on the antibody quality may instruct chimeric SVP designs to optimize immunological outcomes and vaccine efficacy [195]. The RTS,S/AS01 vaccine (Mosquirix™) is the most advanced vaccine with a heterologous antigenic sequence arrayed on SVPs. The RTS,S vaccine is based on the fusion of a *Plasmodium falciparum* CS polypeptide of 189 aa with selected tandem repeats of B-cell and T-cell epitopes to the HBsAgS N-terminus (RTS) (Figure 1C). The CS-protein is expressed on the *Plasmodium* sporozoite surface, and essential for hepatocyte invasion and for establishing a productive infection, and therefore an important target for the development of a pre-erythrocyte vaccine [205]. The genes for HBsAgS and RTS are integrated into the genome of *S. cerevisiae* and co-expressed at a ratio RTS:HBsAgS of 1:4 to generate non-glycosylated mixed (hybrid) lipoprotein particles [190,191]. The RTS,S vaccine is well-tolerated, safe, and immunogenic, and is considered to be the first advanced vaccine against the pre-erythrocyte stage of the malaria parasite, and induces both anti-HBs and anti-CS protein antibodies. The anti-malaria RTS,S/AS01 vaccine in children of five months or older reduced clinical malaria episodes by 39% and life-threatening severe malaria episodes by 29%. The vaccine is licensed in three African countries [5,6,206]. Mosquirix™ (RTS,S/AS01) is adjuvanted with AS01, which is a liposome formulation and contains monophosphoryl lipid A (MPL) and the saponin QS-21. RTS,S in combination with AS01 resulted in higher anti-CS protein immune responses than AS02, which is an oil-in-water emulsion-based adjuvant [7,205,206,207]. To enhance the vaccine efficacy against malaria, a RTS,S-related vaccine (R21) with an increased proportion of CS-polypeptides was developed. CS-polypeptide-HBsAg fusion proteins were expressed in *Pichia pastoris*, and SVPs were obtained after caesium chloride density ultracentrifugation and gel filtration [208]. The R21 vaccine induces a sterile protection in mice against a challenge with transgenic sporozoites. The induction of anti-HBs antibodies is compromised, possibly because the high content of the CS-polypeptide blocks access to the HBsAg external loop region, which contains the antigenic “a”-determinant [208].

Preexisting immunity against vaccine vectors can impose a negative effect on the outcome of the vaccination [210,211,212,213]. HBsAgS SVPs are widely used in immunization programs to combat HBV, and therefore, the use of chimeric SVPs could pose a problem for recipients previously immunized against hepatitis B. Immunization studies in mice using chimeric SVPs with a foreign epitope inserted into the external loop of HBsAgS or fused to the N-terminus of HBsAgS demonstrated that pre-existing anti-HBs antibodies do not compromise the immunogenicity of the foreign antigenic sequence presented by the chimeric HBsAgS SVPs [214,215]. Consistently, clinical studies with human volunteers to assess the RTS,S malaria vaccine did not provide any evidence that a pre-existing anti-HBs status prevented an anti-CS-protein immune response [190]. In relation to a HBV chronic carrier status and the use of the RTS,S vaccine, there was no evidence that chronic HBV carriers (HBsAg positive) and HBsAg-negative individuals respond differently regarding an antibody response to the CS-protein [216].

## 9. Enhancement of Platform Immunogenicity through Biochemical Modifications

Based on the importance of SVPs as medical tools and platforms for the presentation of native viral antigenic sequences, it is critical to understand their immunogenicity in relation to antigen structure in order to enhance or to modulate their immunogenicity. Depending on the SVP type, targeted biochemical modifications of the SVP subunits may allow the generation of SVP variants with enhanced immunogenicity. HBsAgS SVPs are glycosylated lipoprotein particles and are stabilized by extensive intra- and inter-molecular disulfide bonds, which allows targeted modifications of the glycan content and level of disulphide bonding.

Changing disulfide bonding impacts on antigen processing and epitope selection by modifying the conformational flexibility [217]. The three-dimensional structure guides processing and presentation of T helper (Th) and CTL epitopes, and subtle changes in antigen structure can modulate T cell responses due to qualitative and quantitative differences in protein processing [217,218,219,220]. Distinct Th cell epitope profiles emerged from human immunodeficiency virus type 1 (HIV-1) gp120 molecules after destabilizing the three-dimensional structure as a consequence of deleted cysteine residues [221]. In an attempt to enhance immunogenicity, HBsAgS SVPs with a reduced level of disulfide bonds were generated. The biochemically modified SVPs showed a higher protease sensitivity, potentially due to introducing structural changes associated with enhanced cellular immunogenicity [65]. Altering SVP structure may represent an attractive strategy to modulate proteolytic sensitivity to influence antigen processing and promoting an enhanced immune response and/or a changed hierarchy of epitope presentation [218,222].

Manipulation of protein glycosylation represents an alternative strategy to promote antigen internalization and antigen presentation via MHC class I and class II molecules to enhance the adaptive immune responses [223,224]. The glycosylation status and glycan density of the immunogen impacts on its interaction with antigen-presenting cells and recognition by lectins [224]. Glycan-mediated interactions with immunocompetent cells impact on protein uptake and can enhance or modulate cell-mediated and humoral immune responses [225,226,227,228,229]. Contrarily, glycans can shield protein epitopes to evade recognition by antibodies and can block antigen processing [230]. Mannosylation provided an efficient strategy to improve uptake and processing of a SVP derived from the rabbit hemorrhagic disease virus [225]. Consistently, mannosylated solid lipid nanoparticles loaded with HBsAg induced stronger cellular responses than nanoparticles devoid of mannose [229]. Mutant HBsAgS subunits with additional *N*-glycosylation sites assembled into hyperglycosylated SVP. Antigenic fingerprints indicated that additional glycans do not extensively shield HBsAg-specific antigenic sites. Immunization studies demonstrated that the hyperglycosylated SVPs induced earlier and longer-lasting antibody responses than hypoglycosylated SVPs or wild type SVPs [231]. The ability of biochemically modified SVPs to promote immune responses possibly due to differences in their glycosylation-related interaction with cells of the innate immune system illustrates approaches for the design of immunogens with superior immunological characteristics.

## 10. Concluding Remarks

The development of preventative vaccines against hepatitis B resulted in remarkable advances in reducing HBV associated liver diseases. However, chronic hepatitis B is still difficult to control due to continuous viral replication driven by the episomal cccDNA present in the nuclei of infected hepatocytes. Novel strategies to generate vaccines based on structurally modified subunits to enhance immunogenicity and/or to modify antigen processing to change the hierarchy of epitope presentation may represent a pathway to overcome chronic viral infections or may complement a vaccine based on native proteins [65,218,224,225,226,231]. HBsAgS SVPs have been used as carrier platforms for various antigenic sequences to induce anti-foreign humoral and cellular immune responses [8,186]. One of the most advanced chimeric vaccines with a foreign antigenic sequence arrayed on a particulate carrier is based on the HBsAgS backbone fused to a *P. falciparum* CS-polypeptide [5,6]. For the design of next generation vaccines with therapeutic capabilities, formulations based on antigen combinations, such as mixtures of HBsAgS SVPs and SVPs composed of the HBV nucleocapsid antigen (HBcAg), may allow the induction of broad CD4 and CD8 T-cell responses suitable for therapeutic outcomes [232]. Alternatively, the assessment of synergistic effects between biochemically modified immunogens and adjuvant compounds possibly represent an avenue for the generation of optimized vaccines and delivery platforms, which may be suitable for therapeutic applications to overcome established chronic infections.

## Figures and Tables

**Table 1:** Key vaccines against HBV utilizing hepatitis B surface antigens.

Source	Cell type	Name	Antigen	Subtype	Manufacturer
**Plasma-derived vaccines**	-	Heptavax-B^®^	HBsAgS	ad	Merck [116]
	-	Hevac B^®^	HBsAgS, -M	ad *and* ay	Pasteur [120,124,125]
	-	GCC VAC	HBsAgS		Green Cross Operation, Osaka [125]
	-	Hepavax-B	HBsAgS		Korean Green Cross [126,127]
	-	Hepaccine B	HBsAgS		Cheil Foods & Chemicals Company [128]
**Recombinant (yeast-derived)**	*Saccharomyces cerevisiae*	Recombivax^®^ HB HB-Vax II^®^	HBsAgS	adw	Merck [129]
	*Saccharomyces cerevisiae*	Engerix-B^®^	HBsAgS	adw	GlaxoSmithKline [129,130]
	*Saccharomyces cerevisiae*	TGP 943™	HBsAgS, -M	adr	Takeda Chemical Industries [131]
	*Saccharomyces cerevisiae*	Euvax B^®^	HBsAgS		LG Chemical Ltd, [130,132]
	*Pichia pastoris*	Shanvac B	HBsAgS	adw2	Shantha Biotechnics [133]
	*Pichia pastoris*	Heberbiovac-HB^®^	HBsAgS	adw2	Heber Biotech S.A., [130]
	*Hansenula polymorpha*	Heplisav-B^®^	HBsAgS	adw	Dynavax Technologies [134]
	*Hansenula polymorpha*	Hepavax-Gene^TM^	HBsAgS	adr	Janssen Pharma [130,135]
**Recombinant (mammalian cell-derived)**	*Chinese hamster ovary cells*	Gen Hevac B^®^	HBsAgS, -M	ayw	Pasteur [120]
	*Chinese hamster ovary cells*	Sci-B-Vac/Bio-Hep-B™/Hepimmune™	HBsAgS, -M, -L	adw	VBI Vaccines [136]
	*Mouse c127 clonal cell line*	AG-3™ (Hepacare/Hepagene™)	HBsAgS, -M, -L	adw & ayw	Medeva [137]

**Table 2:** Selection of chimeric HBsAg SVP platforms and vaccines.

Antigen	Target	Delivery Site	Expression System	Name	Study/Manufacturer
CS-HBsAg/HBsAgS	Malaria	N-terminal	*Saccharomyces cerevisiae*	RTS,S/AS01 Mosquirix™	GlaxoSmithKline [5,6,190]
CS-HBsAgS	Malaria	N-terminal	*Pichia pastoris*	R21	[208]
DENV-EDIII-HBsAgS/HBsAgS	Dengue virus	N-terminal	*Pichia pastoris*	DSV4	[188]
HBsAgS-gp120 (HIV-1)	HIV-1	C-terminal	CV-1 cell line	MR15, MR23	[189]
Env1-, Env2-HBsAgS	Hepatitis C virus	Substitution/N-terminal extension	CHO cells		[192,209]
HBsAgS-NANP repeats	Malaria	Insertion	HEK293F cell line	M-HBsAg-N4, -N9	[195]
HBsAgS-HCV env epitopes	Hepatitis C virus	Insertion	HEK293T cell line		[196,198]
HBsAgS-catalase epitope	*Helicobacter pylori*	Insertion	HuH-7	VLP-KatA	[197]
HBsAgS-VP1 capsid epitope	Poliovirus	Insertion	Mouse L cells	HBsPolioAg	[201,203]
HBsAgS-matrix CTL epitope	Influenza A virus	Insertion/Substitution	HEK293T cell line		[202]
Polyepitope-HBsAgS;	HIV	Substitution preS2 sequence	SW480 cells		[193]

CS—circumsporozoite polypeptide; DENV-EDIII—Dengue virus envelope domain III; env—envelope protein; HCV—hepatitis C virus; HIV—Human immunodeficiency virus; CTL—cytotoxic T lymphocyte. VLP—virus-like particle.
